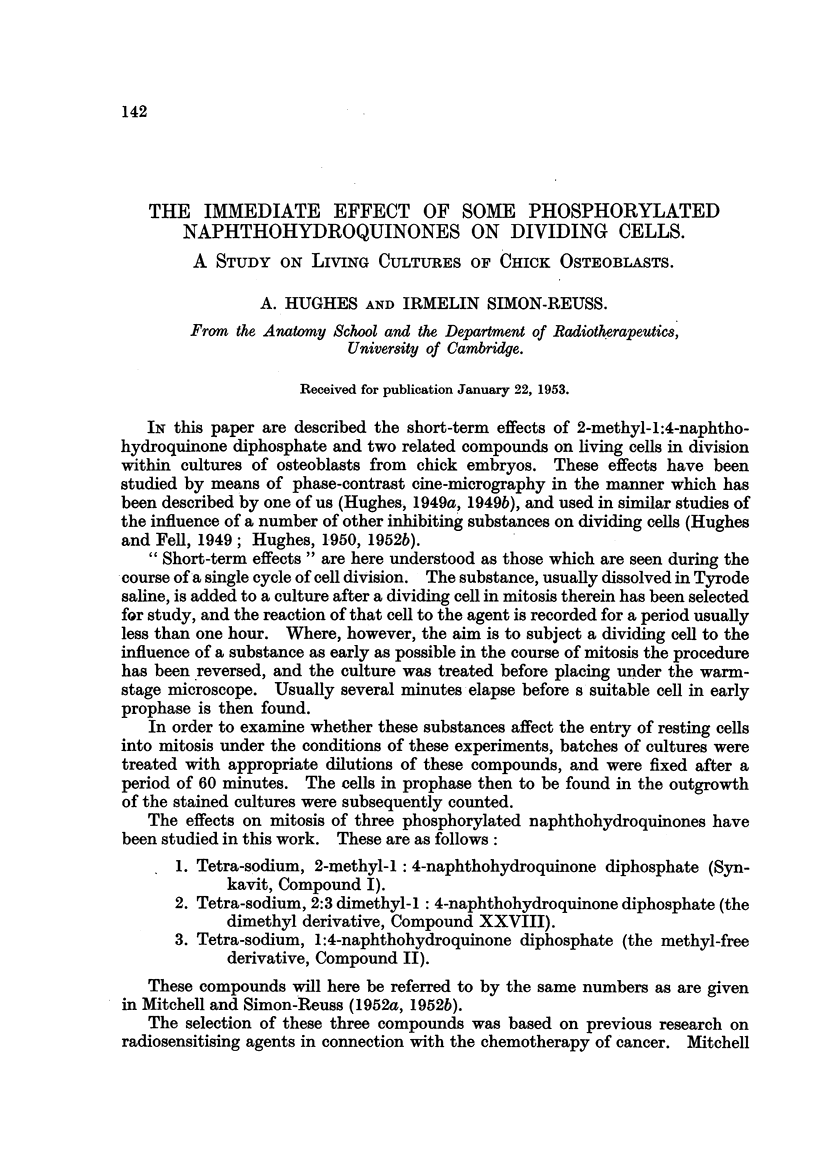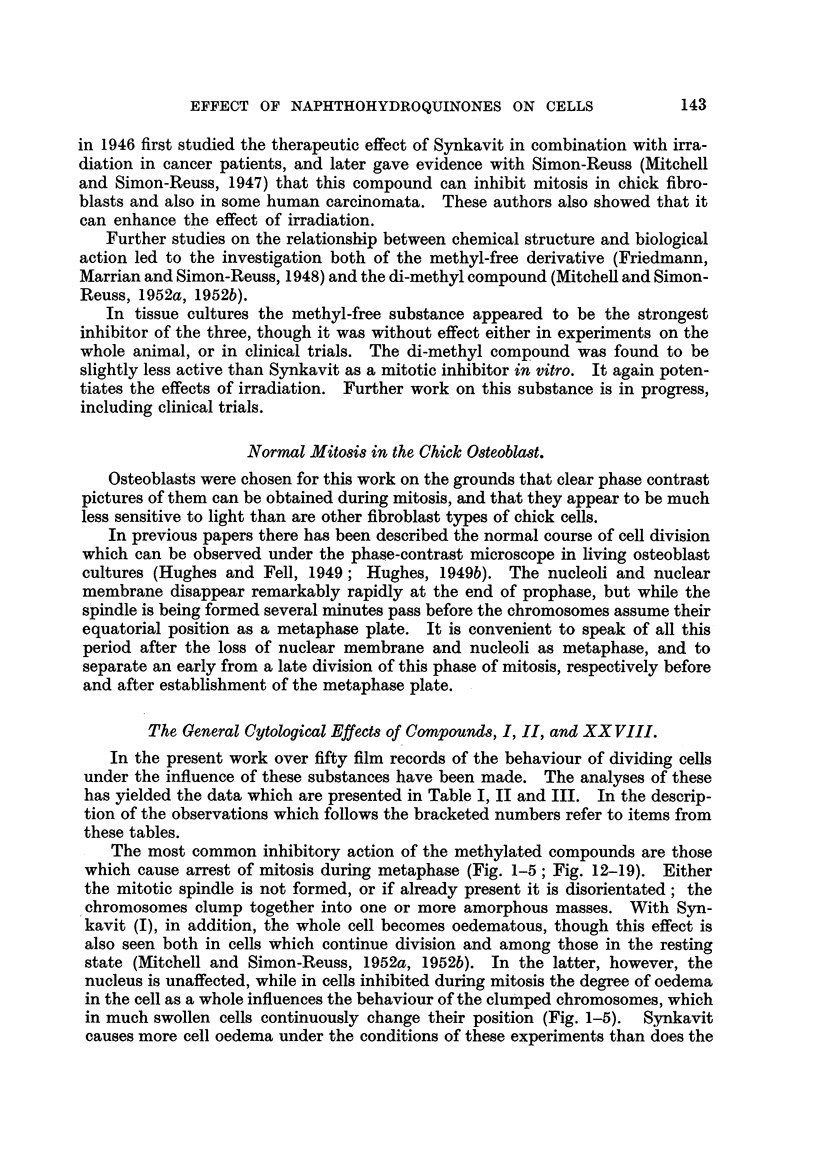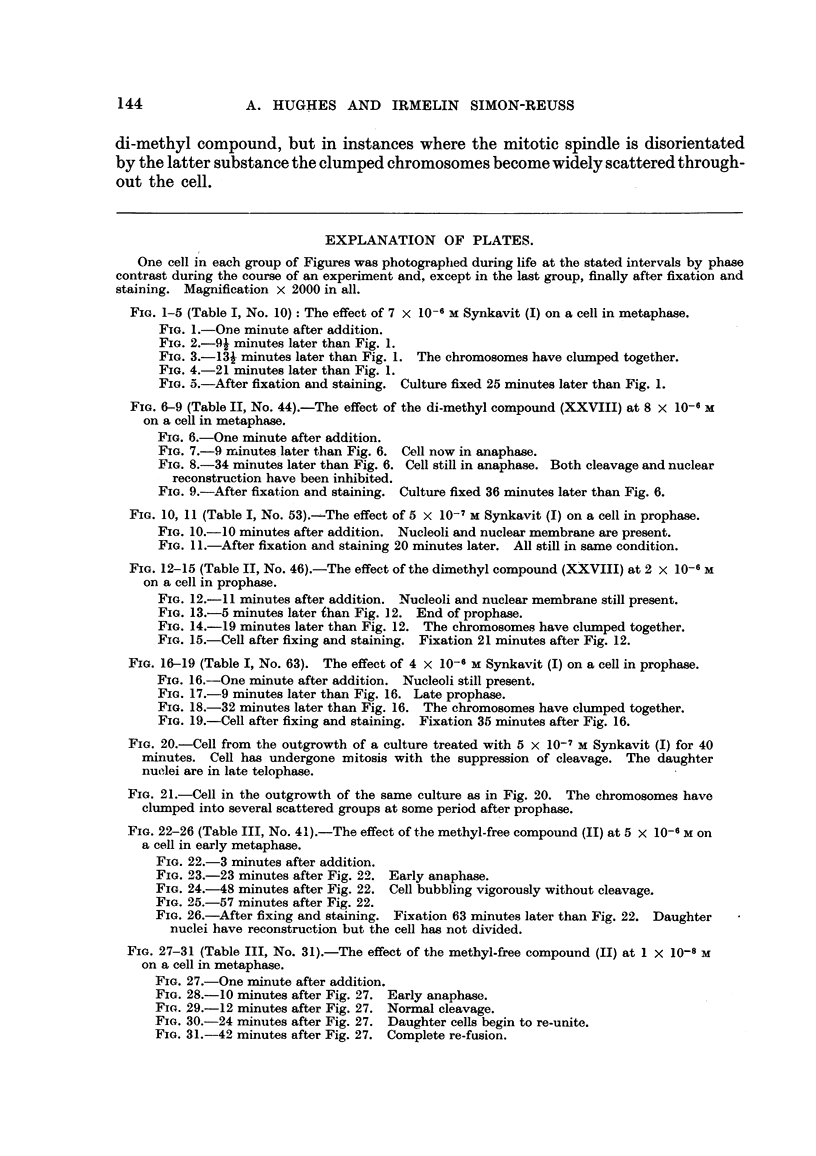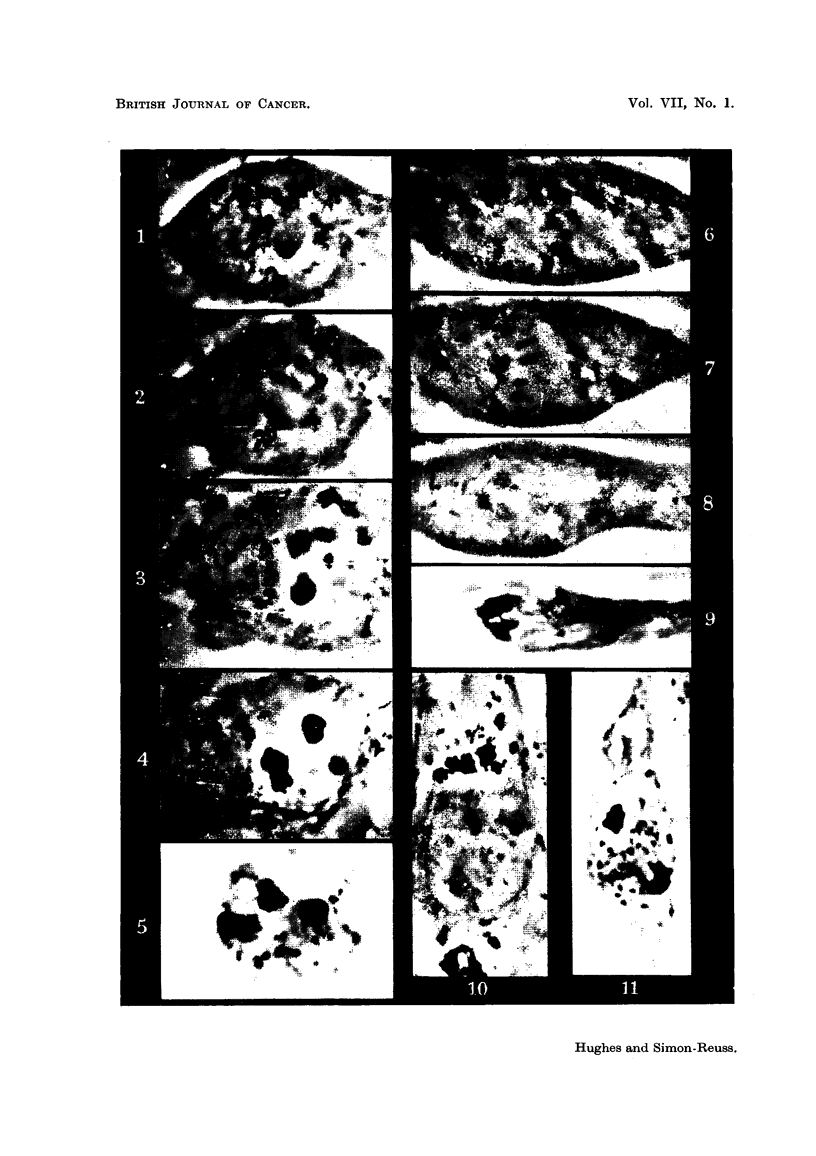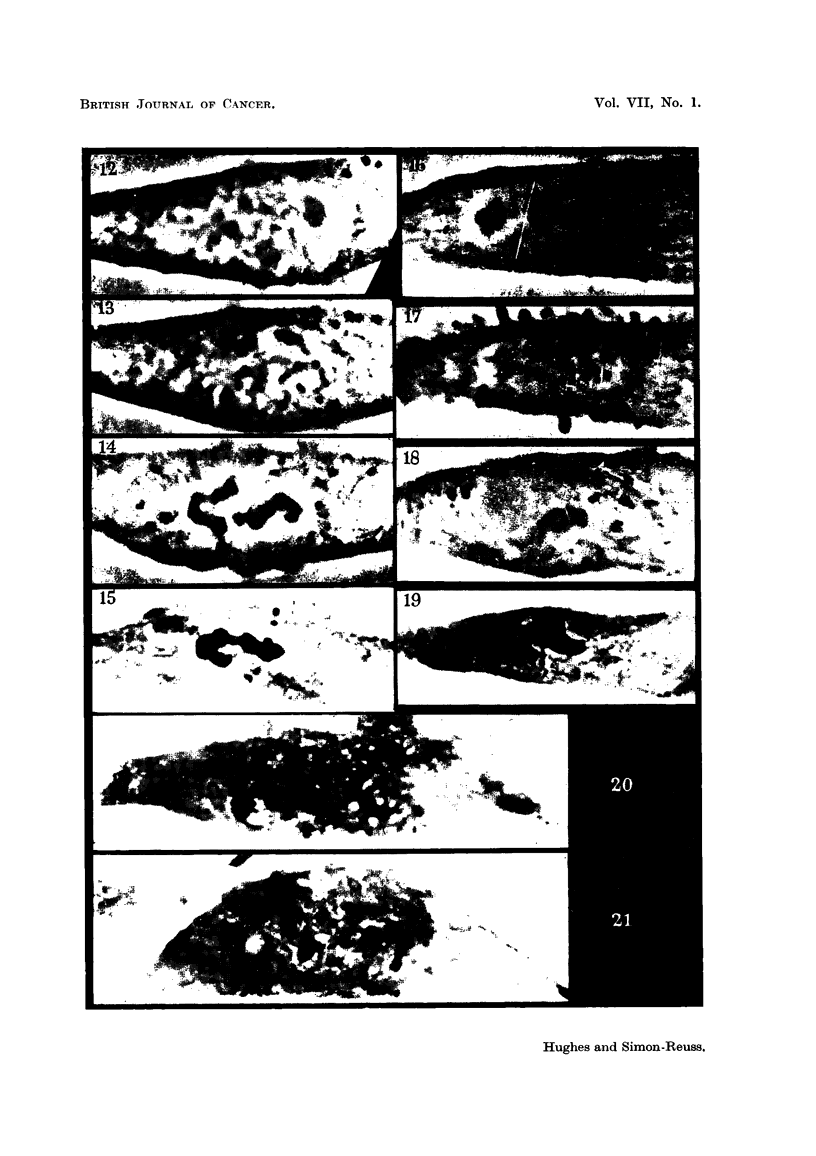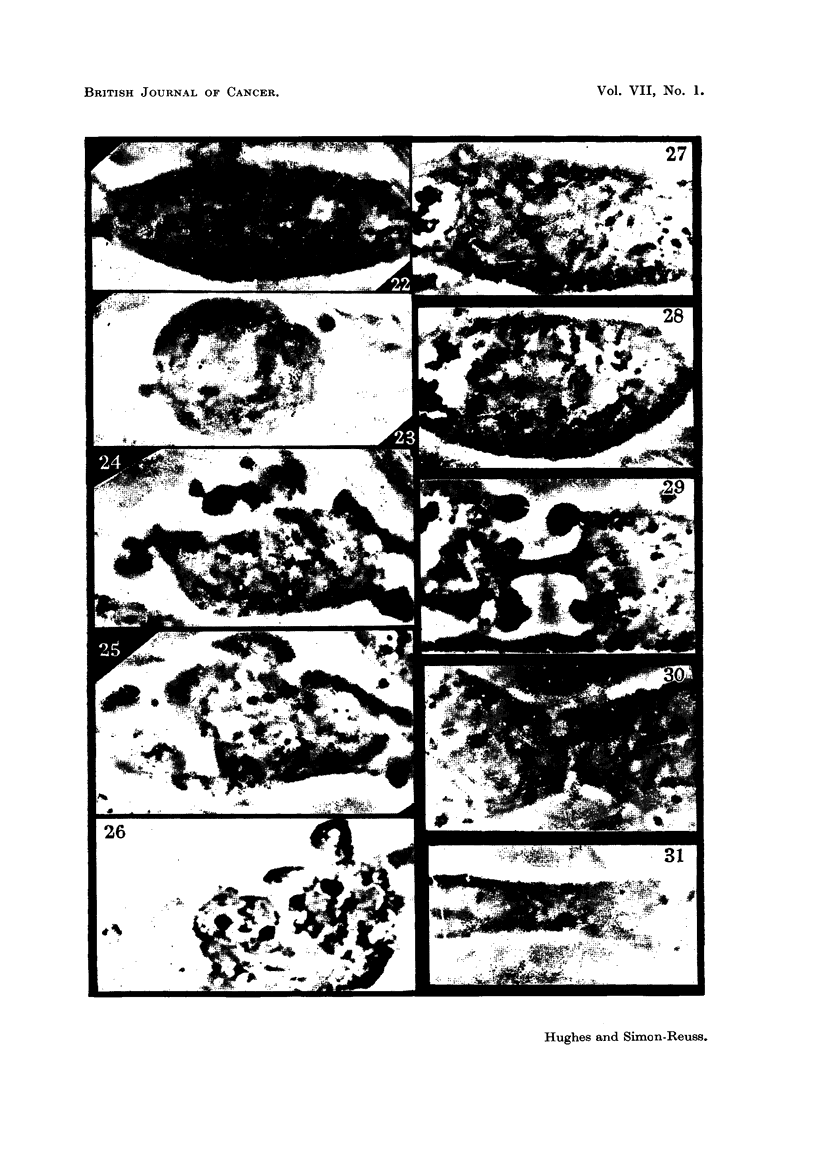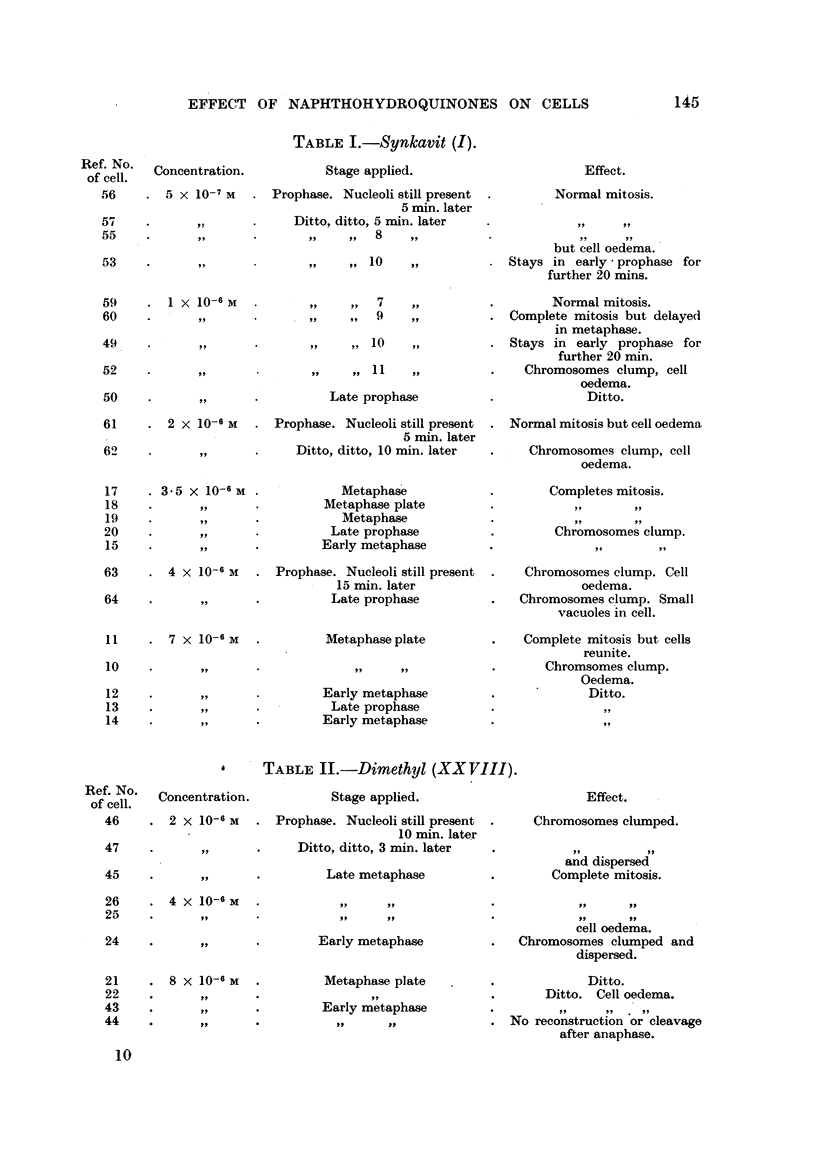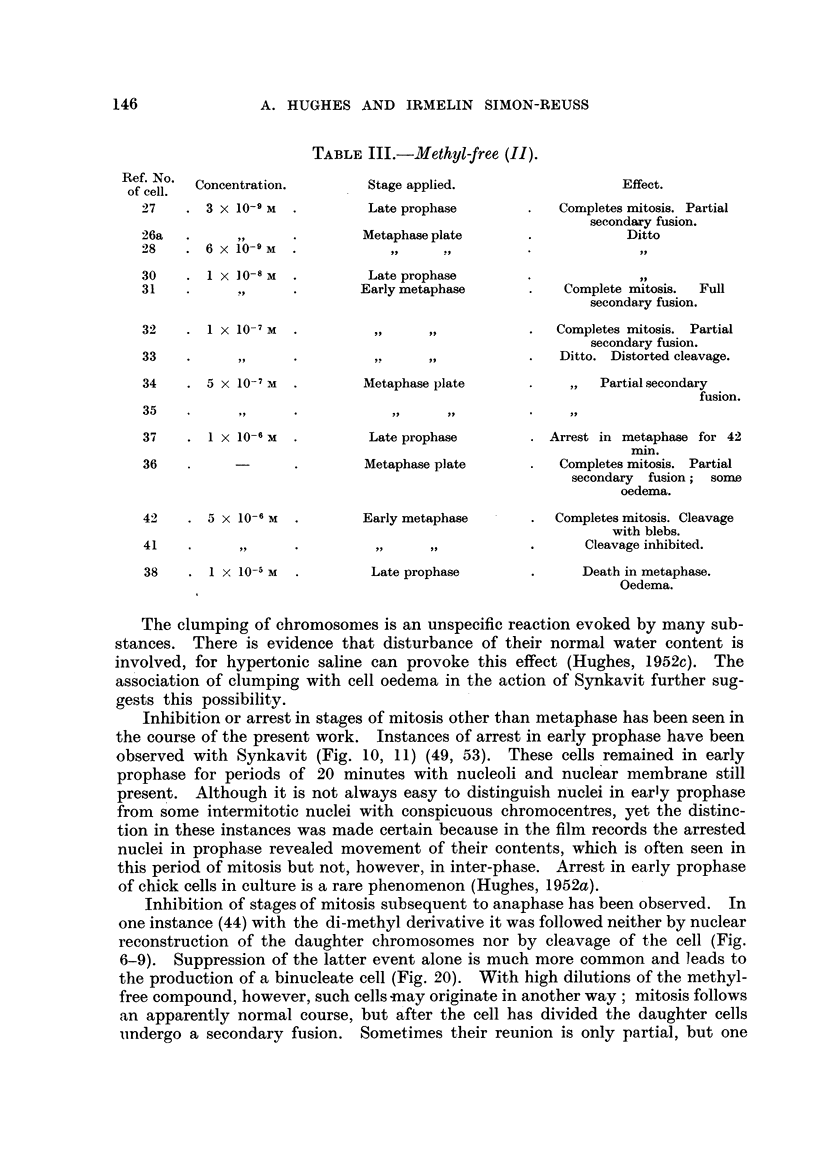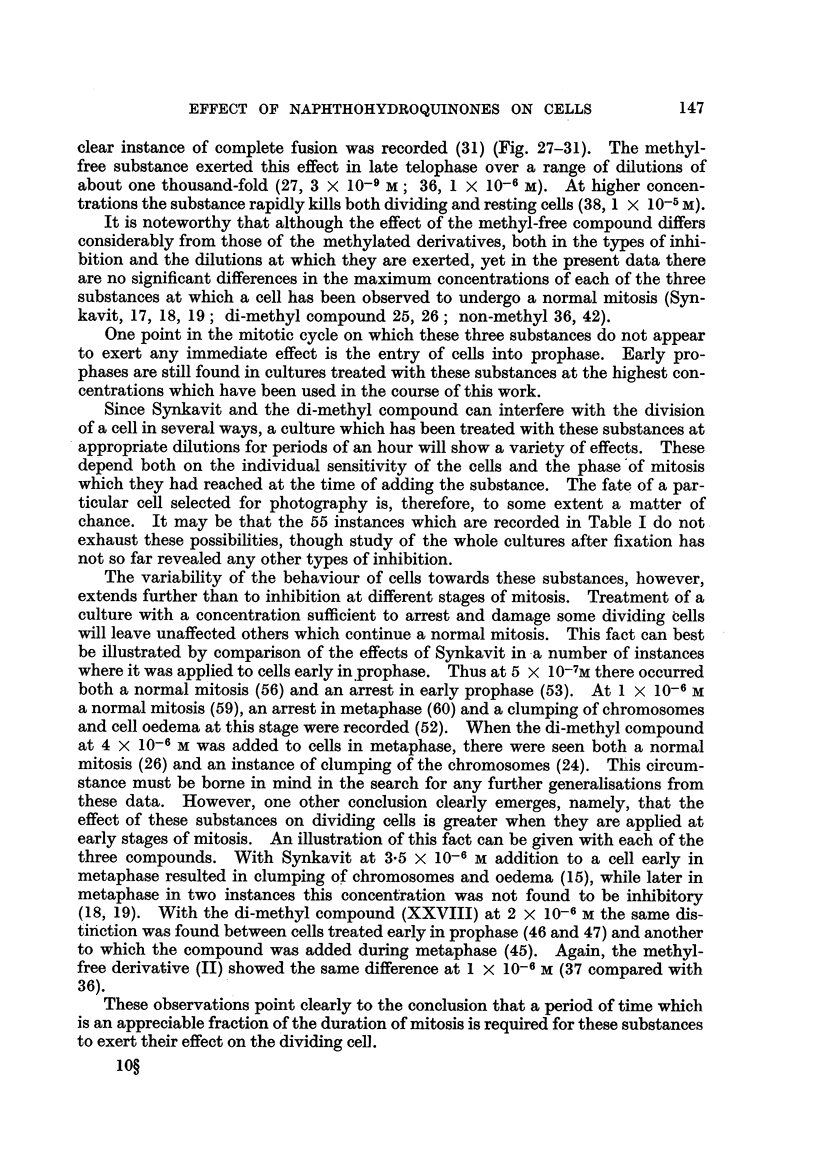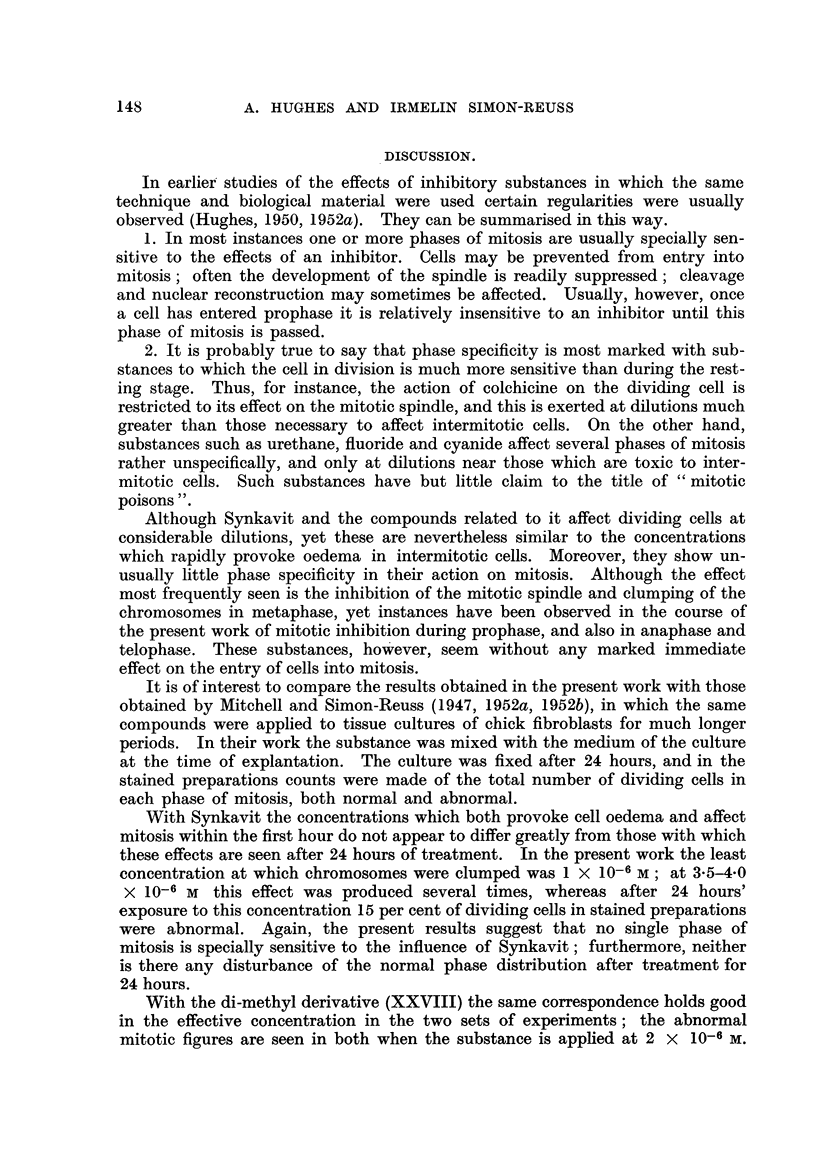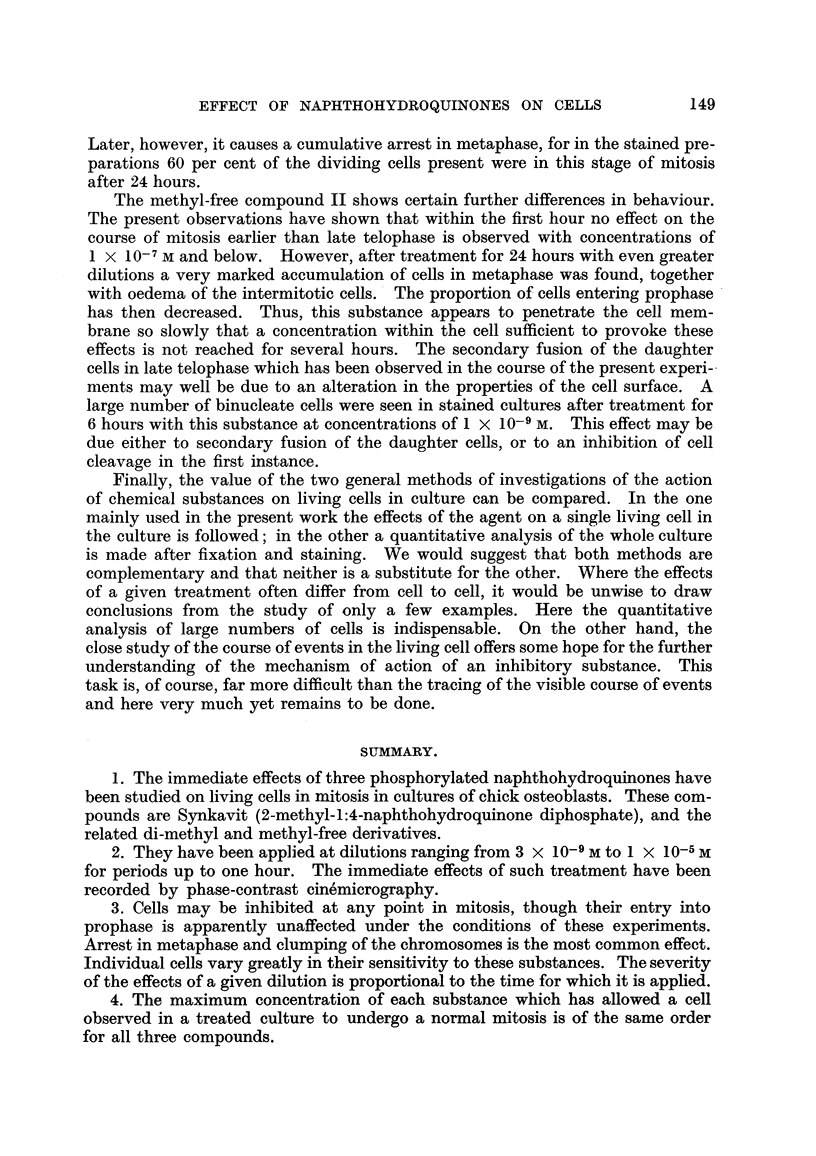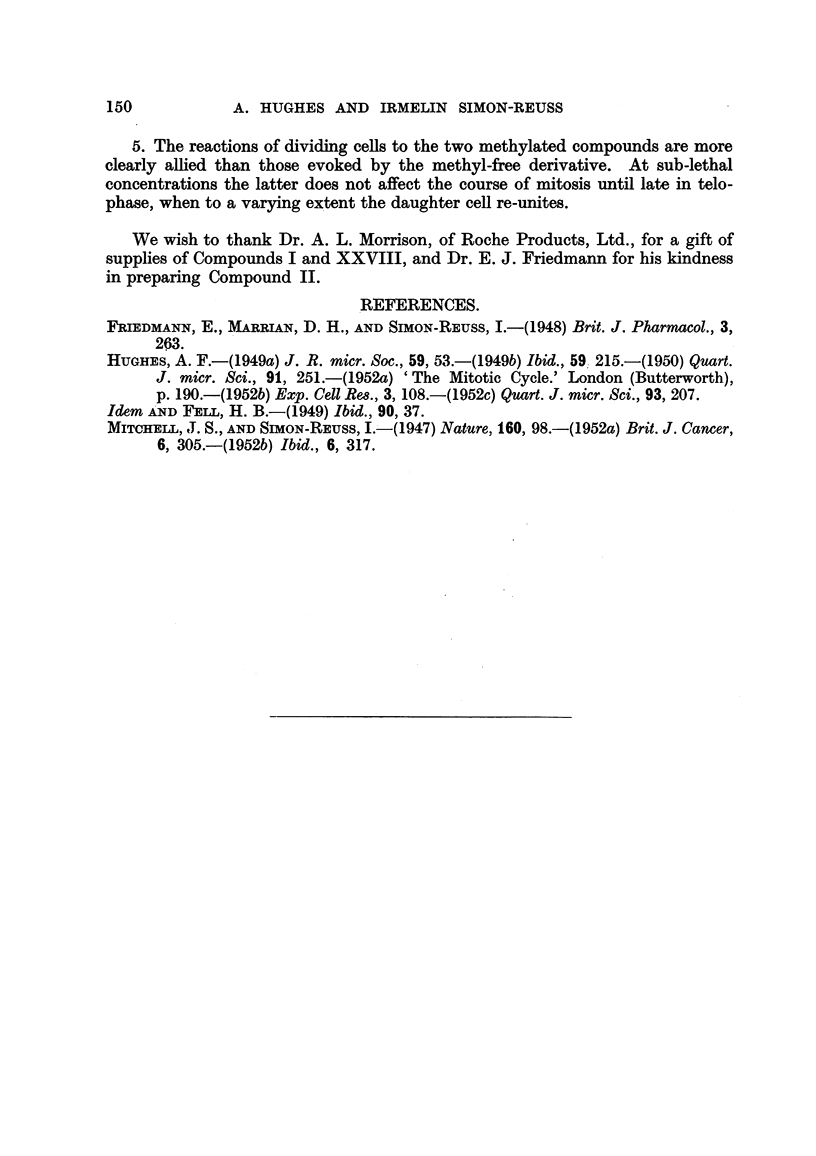# The Immediate Effect of Some Phosphorylated Naphthohydroquinones on Dividing Cells

**DOI:** 10.1038/bjc.1953.13

**Published:** 1953-03

**Authors:** A. Hughes, Irmelin Simon-Reuss

## Abstract

**Images:**


### THE IMMEDIATE EFFECT OF SOME PHOSPHORYLATED NAPHTHOHYDROQUINONES ON DIVIDING CELLS.

### A Study on Living Cultures of Chick Osteoblasts.

A. HUGHES and IRMELIN SIMON-REUSS.

*From the Anatomy School and the Department of Radiotherapeutics, University of Cambridge.*

Received for publication January 22, 1953.

In this paper are described the short-term effects of 2-methyl-1:4-naphthohydroquinone diphosphate and two related compounds on living cells in division within cultures of osteoblasts from chick embryos. These effects have been studied by means of phase-contrast cine-micrography in the manner which has been described by one of us (Hughes, 1949*a*, 1949*b*), and used in similar studies of the influence of a number of other inhibiting substances on dividing cells (Hughes and Fell, 1949 ; Hughes, 1950, 1952*b*).

" Short-term effects " are here understood as those which are seen during the course of a single cycle of cell division. The substance, usually dissolved in Tyrode saline, is added to a culture after a dividing cell in mitosis therein has been selected for study, and the reaction of that cell to the agent is recorded for a period usually less than one hour. Where, however, the aim is to subject a dividing cell to the influence of a substance as early as possible in the course of mitosis the procedure has been reversed, and the culture was treated before placing under the warm-stage microscope. Usually several minutes elapse before s suitable cell in early prophase is then found.

In order to examine whether these substances affect the entry of resting cells into mitosis under the conditions of these experiments, batches of cultures were treated with appropriate dilutions of these compounds, and were fixed after a period of 60 minutes. The cells in prophase then to be found in the outgrowth of the stained cultures were subsequently counted.

The effects on mitosis of three phosphorylated naphthohydroquinones have been studied in this work. These are as follows :

1. Tetra-sodium, 2-methyl-1 : 4-naphthohydroquinone diphosphate (Synkavit, Compound I).
2. Tetra-sodium, 2:3 dimethyl-1 : 4-naphthohydroquinone diphosphate (the dimethyl derivative, Compound XXVIII).
3. Tetra-sodium, 1:4-naphthohydroquinone diphosphate (the methyl-free derivative, Compound II).

These compounds will here be referred to by the same numbers as are given in Mitchell and Simon-Reuss (1952*a*, 1952*b*).

The selection of these three compounds was based on previous research on radiosensitising agents in connection with the chemotherapy of cancer. Mitchell in 1946 first studied the therapeutic effect of Synkavit in combination with irradiation in cancer patients, and later gave evidence with Simon-Reuss (Mitchell and Simon-Reuss, 1947) that this compound can inhibit mitosis in chick fibroblasts and also in some human carcinomata. These authors also showed that it can enhance the effect of irradiation.

Further studies on the relationship between chemical structure and biological action led to the investigation both of the methyl-free derivative (Friedmann, Marrian and Simon-Reuss, 1948) and the di-methyl compound (Mitchell and Simon-Reuss, 1952*a*, 1952*b*).

In tissue cultures the methyl-free substance appeared to be the strongest inhibitor of the three, though it was without effect either in experiments on the whole animal, or in clinical trials. The di-methyl compound was found to be slightly less active than Synkavit as a mitotic inhibitor *in vitro*. It again potentiates the effects of irradiation. Further work on this substance is in progress, including clinical trials.

### *Normal Mitosis in the Chick Osteoblast.*

Osteoblasts were chosen for this work on the grounds that clear phase contrast pictures of them can be obtained during mitosis, and that they appear to be much less sensitive to light than are other fibroblast types of chick cells.

In previous papers there has been described the normal course of cell division which can be observed under the phase-contrast microscope in living osteoblast cultures (Hughes and Fell, 1949; Hughes, 1949*b*). The nucleoli and nuclear membrane disappear remarkably rapidly at the end of prophase, but while the spindle is being formed several minutes pass before the chromosomes assume their equatorial position as a metaphase plate. It is convenient to speak of all this period after the loss of nuclear membrane and nucleoli as metaphase, and to separate an early from a late division of this phase of mitosis, respectively before and after establishment of the metaphase plate.

### *The General Cytological Effects of Compounds, I, II, and XXVIII.*

In the present work over fifty film records of the behaviour of dividing cells under the influence of these substances have been made. The analyses of these has yielded the data which are presented in Table I, II and III. In the description of the observations which follows the bracketed numbers refer to items from these tables.

The most common inhibitory action of the methylated compounds are those which cause arrest of mitosis during metaphase (Fig. 1–5; Fig. 12–19). Either the mitotic spindle is not formed, or if already present it is disorientated; the chromosomes clump together into one or more amorphous masses. With Synkavit (I), in addition, the whole cell becomes oedematous, though this effect is also seen both in cells which continue division and among those in the resting state (Mitchell and Simon-Reuss, 1952*a*, 1952*b*). In the latter, however, the nucleus is unaffected, while in cells inhibited during mitosis the degree of oedema in the cell as a whole influences the behaviour of the clumped chromosomes, which in much swollen cells continuously change their position (Fig. 1–5). Synkavit causes more cell oedema under the conditions of these experiments than does the di-methyl compound, but in instances where the mitotic spindle is disorientated by the latter substance the clumped chromosomes become widely scattered throughout the cell.

---

### EXPLANATION OF PLATES.

One cell in each group of Figures was photographed during life at the stated intervals by phase contrast during the course of an experiment and, except in the last group, finally after fixation and staining. Magnification $\times$ 2000 in all.

FIG. 1–5 (Table I, No. 10) : The effect of 7 $\times$ $10^{-6}$ M Synkavit (I) on a cell in metaphase.

FIG. 1.—One minute after addition.
FIG. 2.—9½ minutes later than Fig. 1.
FIG. 3.—13½ minutes later than Fig. 1. The chromosomes have clumped together.
FIG. 4.—21 minutes later than Fig. 1.
FIG. 5.—After fixation and staining. Culture fixed 25 minutes later than Fig. 1.

FIG. 6–9 (Table II, No. 44).—The effect of the di-methyl compound (XXVIII) at 8 $\times$ $10^{-6}$ M on a cell in metaphase.

FIG. 6.—One minute after addition.
FIG. 7.—9 minutes later than Fig. 6. Cell now in anaphase.
FIG. 8.—34 minutes later than Fig. 6. Cell still in anaphase. Both cleavage and nuclear reconstruction have been inhibited.
FIG. 9.—After fixation and staining. Culture fixed 36 minutes later than Fig. 6.

FIG. 10, 11 (Table I, No. 53).—The effect of 5 $\times$ $10^{-7}$ M Synkavit (I) on a cell in prophase.

FIG. 10.—10 minutes after addition. Nucleoli and nuclear membrane are present.
FIG. 11.—After fixation and staining 20 minutes later. All still in same condition.

FIG. 12–15 (Table II, No. 46).—The effect of the dimethyl compound (XXVIII) at 2 $\times$ $10^{-6}$ M on a cell in prophase.

FIG. 12.—11 minutes after addition. Nucleoli and nuclear membrane still present.
FIG. 13.—5 minutes later than Fig. 12. End of prophase.
FIG. 14.—19 minutes later than Fig. 12. The chromosomes have clumped together.
FIG. 15.—Cell after fixing and staining. Fixation 21 minutes after Fig. 12.

FIG. 16–19 (Table I, No. 63). The effect of 4 $\times$ $10^{-6}$ M Synkavit (I) on a cell in prophase.

FIG. 16.—One minute after addition. Nucleoli still present.
FIG. 17.—9 minutes later than Fig. 16. Late prophase.
FIG. 18.—32 minutes later than Fig. 16. The chromosomes have clumped together.
FIG. 19.—Cell after fixing and staining. Fixation 35 minutes after Fig. 16.

FIG. 20.—Cell from the outgrowth of a culture treated with 5 $\times$ $10^{-7}$ M Synkavit (I) for 40 minutes. Cell has undergone mitosis with the suppression of cleavage. The daughter nuclei are in late telophase.

FIG. 21.—Cell in the outgrowth of the same culture as in Fig. 20. The chromosomes have clumped into several scattered groups at some period after prophase.

FIG. 22–26 (Table III, No. 41).—The effect of the methyl-free compound (II) at 5 $\times$ $10^{-6}$ M on a cell in early metaphase.

FIG. 22.—3 minutes after addition.
FIG. 23.—23 minutes after Fig. 22. Early anaphase.
FIG. 24.—48 minutes after Fig. 22. Cell bubbling vigorously without cleavage.
FIG. 25.—57 minutes after Fig. 22.
FIG. 26.—After fixing and staining. Fixation 63 minutes later than Fig. 22. Daughter nuclei have reconstruction but the cell has not divided.

FIG. 27–31 (Table III, No. 31).—The effect of the methyl-free compound (II) at 1 $\times$ $10^{-8}$ M on a cell in metaphase.

FIG. 27.—One minute after addition.
FIG. 28.—10 minutes after Fig. 27. Early anaphase.
FIG. 29.—12 minutes after Fig. 27. Normal cleavage.
FIG. 30.—24 minutes after Fig. 27. Daughter cells begin to re-unite.
FIG. 31.—42 minutes after Fig. 27. Complete re-fusion.

1
2
3
4
5
6
7
8
9
10
11

Hughes and Simon-Reuss.

Hughes and Simon-Reuss.

### Table I.—*Synkavit* (*I*).

| Ref. No. of cell. | Concentration. | Stage applied. | Effect. |
|---|---|---|---|
| 56 | $5 \times 10^{-7}$ M | Prophase. Nucleoli still present 5 min. later | Normal mitosis. |
| 57 | " | Ditto, ditto, 5 min. later | " " |
| 55 | " | " " 8 " | " " but cell oedema. |
| 53 | " | " " 10 " | Stays in early prophase for further 20 mins. |
| 59 | $1 \times 10^{-6}$ M | " " 7 " | Normal mitosis. |
| 60 | " | " " 9 " | Complete mitosis but delayed in metaphase. |
| 49 | " | " " 10 " | Stays in early prophase for further 20 min. |
| 52 | " | " " 11 " | Chromosomes clump, cell oedema. |
| 50 | " | Late prophase | Ditto. |
| 61 | $2 \times 10^{-6}$ M | Prophase. Nucleoli still present 5 min. later | Normal mitosis but cell oedema |
| 62 | " | Ditto, ditto, 10 min. later | Chromosomes clump, cell oedema. |
| 17 | $3{\cdot}5 \times 10^{-6}$ M | Metaphase | Completes mitosis. |
| 18 | " | Metaphase plate | " " |
| 19 | " | Metaphase | " " |
| 20 | " | Late prophase | Chromosomes clump. |
| 15 | " | Early metaphase | " " |
| 63 | $4 \times 10^{-6}$ M | Prophase. Nucleoli still present 15 min. later | Chromosomes clump. Cell oedema. |
| 64 | " | Late prophase | Chromosomes clump. Small vacuoles in cell. |
| 11 | $7 \times 10^{-6}$ M | Metaphase plate | Complete mitosis but cells reunite. |
| 10 | " | " " | Chromsomes clump. Oedema. |
| 12 | " | Early metaphase | Ditto. |
| 13 | " | Late prophase | " |
| 14 | " | Early metaphase | " |

### Table II.—*Dimethyl* (*XXVIII*).

| Ref. No. of cell. | Concentration. | Stage applied. | Effect. |
|---|---|---|---|
| 46 | $2 \times 10^{-6}$ M | Prophase. Nucleoli still present 10 min. later | Chromosomes clumped. |
| 47 | " | Ditto, ditto, 3 min. later | " " and dispersed |
| 45 | " | Late metaphase | Complete mitosis. |
| 26 | $4 \times 10^{-6}$ M | " " | " " |
| 25 | " | " " | " " cell oedema. |
| 24 | " | Early metaphase | Chromosomes clumped and dispersed. |
| 21 | $8 \times 10^{-6}$ M | Metaphase plate | Ditto. |
| 22 | " | " | Ditto. Cell oedema. |
| 43 | " | Early metaphase | " " " |
| 44 | " | " " | No reconstruction or cleavage after anaphase. |

TABLE III.—*Methyl-free (II)*.

| Ref. No. of cell. | Concentration. | Stage applied. | Effect. |
|---|---|---|---|
| 27 | $3 \times 10^{-9}$ M | Late prophase | Completes mitosis. Partial secondary fusion. |
| 26a | „ | Metaphase plate | Ditto |
| 28 | $6 \times 10^{-9}$ M | „ „ | „ |
| 30 | $1 \times 10^{-8}$ M | Late prophase | „ |
| 31 | „ | Early metaphase | Complete mitosis. Full secondary fusion. |
| 32 | $1 \times 10^{-7}$ M | „ „ | Completes mitosis. Partial secondary fusion. |
| 33 | „ | „ „ | Ditto. Distorted cleavage. |
| 34 | $5 \times 10^{-7}$ M | Metaphase plate | „ Partial secondary fusion. |
| 35 | „ | „ „ | „ |
| 37 | $1 \times 10^{-6}$ M | Late prophase | Arrest in metaphase for 42 min. |
| 36 | — | Metaphase plate | Completes mitosis. Partial secondary fusion; some oedema. |
| 42 | $5 \times 10^{-6}$ M | Early metaphase | Completes mitosis. Cleavage with blebs. |
| 41 | „ | „ „ | Cleavage inhibited. |
| 38 | $1 \times 10^{-5}$ M | Late prophase | Death in metaphase. Oedema. |

The clumping of chromosomes is an unspecific reaction evoked by many substances. There is evidence that disturbance of their normal water content is involved, for hypertonic saline can provoke this effect (Hughes, 1952*c*). The association of clumping with cell oedema in the action of Synkavit further suggests this possibility.

Inhibition or arrest in stages of mitosis other than metaphase has been seen in the course of the present work. Instances of arrest in early prophase have been observed with Synkavit (Fig. 10, 11) (49, 53). These cells remained in early prophase for periods of 20 minutes with nucleoli and nuclear membrane still present. Although it is not always easy to distinguish nuclei in early prophase from some intermitotic nuclei with conspicuous chromocentres, yet the distinction in these instances was made certain because in the film records the arrested nuclei in prophase revealed movement of their contents, which is often seen in this period of mitosis but not, however, in inter-phase. Arrest in early prophase of chick cells in culture is a rare phenomenon (Hughes, 1952*a*).

Inhibition of stages of mitosis subsequent to anaphase has been observed. In one instance (44) with the di-methyl derivative it was followed neither by nuclear reconstruction of the daughter chromosomes nor by cleavage of the cell (Fig. 6–9). Suppression of the latter event alone is much more common and leads to the production of a binucleate cell (Fig. 20). With high dilutions of the methyl-free compound, however, such cells may originate in another way ; mitosis follows an apparently normal course, but after the cell has divided the daughter cells undergo a secondary fusion. Sometimes their reunion is only partial, but one clear instance of complete fusion was recorded (31) (Fig. 27–31). The methyl-free substance exerted this effect in late telophase over a range of dilutions of about one thousand-fold (27, $3 \times 10^{-9}$ M; 36, $1 \times 10^{-6}$ M). At higher concentrations the substance rapidly kills both dividing and resting cells (38, $1 \times 10^{-5}$ M).

It is noteworthy that although the effect of the methyl-free compound differs considerably from those of the methylated derivatives, both in the types of inhibition and the dilutions at which they are exerted, yet in the present data there are no significant differences in the maximum concentrations of each of the three substances at which a cell has been observed to undergo a normal mitosis (Synkavit, 17, 18, 19; di-methyl compound 25, 26; non-methyl 36, 42).

One point in the mitotic cycle on which these three substances do not appear to exert any immediate effect is the entry of cells into prophase. Early prophases are still found in cultures treated with these substances at the highest concentrations which have been used in the course of this work.

Since Synkavit and the di-methyl compound can interfere with the division of a cell in several ways, a culture which has been treated with these substances at appropriate dilutions for periods of an hour will show a variety of effects. These depend both on the individual sensitivity of the cells and the phase of mitosis which they had reached at the time of adding the substance. The fate of a particular cell selected for photography is, therefore, to some extent a matter of chance. It may be that the 55 instances which are recorded in Table I do not exhaust these possibilities, though study of the whole cultures after fixation has not so far revealed any other types of inhibition.

The variability of the behaviour of cells towards these substances, however, extends further than to inhibition at different stages of mitosis. Treatment of a culture with a concentration sufficient to arrest and damage some dividing cells will leave unaffected others which continue a normal mitosis. This fact can best be illustrated by comparison of the effects of Synkavit in a number of instances where it was applied to cells early in prophase. Thus at $5 \times 10^{-7}$M there occurred both a normal mitosis (56) and an arrest in early prophase (53). At $1 \times 10^{-6}$ M a normal mitosis (59), an arrest in metaphase (60) and a clumping of chromosomes and cell oedema at this stage were recorded (52). When the di-methyl compound at $4 \times 10^{-6}$ M was added to cells in metaphase, there were seen both a normal mitosis (26) and an instance of clumping of the chromosomes (24). This circumstance must be borne in mind in the search for any further generalisations from these data. However, one other conclusion clearly emerges, namely, that the effect of these substances on dividing cells is greater when they are applied at early stages of mitosis. An illustration of this fact can be given with each of the three compounds. With Synkavit at $3{\cdot}5 \times 10^{-6}$ M addition to a cell early in metaphase resulted in clumping of chromosomes and oedema (15), while later in metaphase in two instances this concentration was not found to be inhibitory (18, 19). With the di-methyl compound (XXVIII) at $2 \times 10^{-6}$ M the same distinction was found between cells treated early in prophase (46 and 47) and another to which the compound was added during metaphase (45). Again, the methyl-free derivative (II) showed the same difference at $1 \times 10^{-6}$ M (37 compared with 36).

These observations point clearly to the conclusion that a period of time which is an appreciable fraction of the duration of mitosis is required for these substances to exert their effect on the dividing cell.

### DISCUSSION.

In earlier studies of the effects of inhibitory substances in which the same technique and biological material were used certain regularities were usually observed (Hughes, 1950, 1952*a*). They can be summarised in this way.

1. In most instances one or more phases of mitosis are usually specially sensitive to the effects of an inhibitor. Cells may be prevented from entry into mitosis; often the development of the spindle is readily suppressed; cleavage and nuclear reconstruction may sometimes be affected. Usually, however, once a cell has entered prophase it is relatively insensitive to an inhibitor until this phase of mitosis is passed.

2. It is probably true to say that phase specificity is most marked with substances to which the cell in division is much more sensitive than during the resting stage. Thus, for instance, the action of colchicine on the dividing cell is restricted to its effect on the mitotic spindle, and this is exerted at dilutions much greater than those necessary to affect intermitotic cells. On the other hand, substances such as urethane, fluoride and cyanide affect several phases of mitosis rather unspecifically, and only at dilutions near those which are toxic to intermitotic cells. Such substances have but little claim to the title of "mitotic poisons".

Although Synkavit and the compounds related to it affect dividing cells at considerable dilutions, yet these are nevertheless similar to the concentrations which rapidly provoke oedema in intermitotic cells. Moreover, they show unusually little phase specificity in their action on mitosis. Although the effect most frequently seen is the inhibition of the mitotic spindle and clumping of the chromosomes in metaphase, yet instances have been observed in the course of the present work of mitotic inhibition during prophase, and also in anaphase and telophase. These substances, however, seem without any marked immediate effect on the entry of cells into mitosis.

It is of interest to compare the results obtained in the present work with those obtained by Mitchell and Simon-Reuss (1947, 1952*a*, 1952*b*), in which the same compounds were applied to tissue cultures of chick fibroblasts for much longer periods. In their work the substance was mixed with the medium of the culture at the time of explantation. The culture was fixed after 24 hours, and in the stained preparations counts were made of the total number of dividing cells in each phase of mitosis, both normal and abnormal.

With Synkavit the concentrations which both provoke cell oedema and affect mitosis within the first hour do not appear to differ greatly from those with which these effects are seen after 24 hours of treatment. In the present work the least concentration at which chromosomes were clumped was $1 \times 10^{-6}$ M; at 3·5–4·0 $\times 10^{-6}$ M this effect was produced several times, whereas after 24 hours' exposure to this concentration 15 per cent of dividing cells in stained preparations were abnormal. Again, the present results suggest that no single phase of mitosis is specially sensitive to the influence of Synkavit; furthermore, neither is there any disturbance of the normal phase distribution after treatment for 24 hours.

With the di-methyl derivative (XXVIII) the same correspondence holds good in the effective concentration in the two sets of experiments; the abnormal mitotic figures are seen in both when the substance is applied at $2 \times 10^{-6}$ M.

Later, however, it causes a cumulative arrest in metaphase, for in the stained preparations 60 per cent of the dividing cells present were in this stage of mitosis after 24 hours.

The methyl-free compound II shows certain further differences in behaviour. The present observations have shown that within the first hour no effect on the course of mitosis earlier than late telophase is observed with concentrations of $1 \times 10^{-7}$ M and below. However, after treatment for 24 hours with even greater dilutions a very marked accumulation of cells in metaphase was found, together with oedema of the intermitotic cells. The proportion of cells entering prophase has then decreased. Thus, this substance appears to penetrate the cell membrane so slowly that a concentration within the cell sufficient to provoke these effects is not reached for several hours. The secondary fusion of the daughter cells in late telophase which has been observed in the course of the present experiments may well be due to an alteration in the properties of the cell surface. A large number of binucleate cells were seen in stained cultures after treatment for 6 hours with this substance at concentrations of $1 \times 10^{-9}$ M. This effect may be due either to secondary fusion of the daughter cells, or to an inhibition of cell cleavage in the first instance.

Finally, the value of the two general methods of investigations of the action of chemical substances on living cells in culture can be compared. In the one mainly used in the present work the effects of the agent on a single living cell in the culture is followed; in the other a quantitative analysis of the whole culture is made after fixation and staining. We would suggest that both methods are complementary and that neither is a substitute for the other. Where the effects of a given treatment often differ from cell to cell, it would be unwise to draw conclusions from the study of only a few examples. Here the quantitative analysis of large numbers of cells is indispensable. On the other hand, the close study of the course of events in the living cell offers some hope for the further understanding of the mechanism of action of an inhibitory substance. This task is, of course, far more difficult than the tracing of the visible course of events and here very much yet remains to be done.

### SUMMARY.

1. The immediate effects of three phosphorylated naphthohydroquinones have been studied on living cells in mitosis in cultures of chick osteoblasts. These compounds are Synkavit (2-methyl-1:4-naphthohydroquinone diphosphate), and the related di-methyl and methyl-free derivatives.

2. They have been applied at dilutions ranging from $3 \times 10^{-9}$ M to $1 \times 10^{-5}$ M for periods up to one hour. The immediate effects of such treatment have been recorded by phase-contrast cinémicrography.

3. Cells may be inhibited at any point in mitosis, though their entry into prophase is apparently unaffected under the conditions of these experiments. Arrest in metaphase and clumping of the chromosomes is the most common effect. Individual cells vary greatly in their sensitivity to these substances. The severity of the effects of a given dilution is proportional to the time for which it is applied.

4. The maximum concentration of each substance which has allowed a cell observed in a treated culture to undergo a normal mitosis is of the same order for all three compounds.

5. The reactions of dividing cells to the two methylated compounds are more clearly allied than those evoked by the methyl-free derivative. At sub-lethal concentrations the latter does not affect the course of mitosis until late in telophase, when to a varying extent the daughter cell re-unites.

We wish to thank Dr. A. L. Morrison, of Roche Products, Ltd., for a gift of supplies of Compounds I and XXVIII, and Dr. E. J. Friedmann for his kindness in preparing Compound II.

### REFERENCES.

Friedmann, E., Marrian, D. H., and Simon-Reuss, I.—(1948) *Brit. J. Pharmacol.*, **3**, 263.

Hughes, A. F.—(1949*a*) *J. R. micr. Soc.*, **59**, 53.—(1949*b*) *Ibid.*, **59** 215.—(1950) *Quart. J. micr. Sci.*, **91**, 251.—(1952*a*) 'The Mitotic Cycle.' London (Butterworth), p. 190.—(1952*b*) *Exp. Cell Res.*, **3**, 108.—(1952*c*) *Quart. J. micr. Sci.*, **93**, 207.

*Idem* and Fell, H. B.—(1949) *Ibid.*, **90**, 37.

Mitchell, J. S., and Simon-Reuss, I.—(1947) *Nature*, **160**, 98.—(1952*a*) *Brit. J. Cancer*, **6**, 305.—(1952*b*) *Ibid.*, **6**, 317.